# Fertility in Patients with Thalassemia and Outcome of Pregnancies: A Turkish Experience

**DOI:** 10.4274/tjh.galenos.2019.2019.0025

**Published:** 2019-11-18

**Authors:** Burcu Akıncı, Akkız Şahin Yaşar, Nihal Özdemir Karadaş, Zuhal Önder Siviş, Hamiyet Hekimci Özdemir, Deniz Yılmaz Karapınar, Can Balkan, Kaan Kavaklı, Yeşim Aydınok

**Affiliations:** 1 Ege University Faculty of Medicine, Department of Pediatric Hematology, Thalassemia Center, İzmir, Turkey

**Keywords:** Thalassemia, Fertility, Pregnancy

## Abstract

**Objective:**

In recent years, the rates of marriage and pregnancy are increasing in patients with thalassemia major. The aim of the present study was to investigate the fertility rate of thalassemic patients and the course of pregnancies in terms of mother and infant health.

**Materials and Methods:**

In this observational study patients with major hemoglobinopathy were evaluated regarding marital status, the need for assisted reproductive techniques, fertility rate, iron status, and pregnancy complications.

**Results:**

Seventeen female patients gave birth to 21 healthy infants. About one-third of the patients needed assisted reproductive techniques. Thalassemia major patients showed increased serum ferritin levels from 1203±1206 μg/L at baseline to 1880±1174 μg/L at the end of pregnancy. All babies are still alive and healthy.

**Conclusion:**

Pregnancy in patients with thalassemia can be safe for the mother and newborn with close monitoring and a multidisciplinary approach.

## Abstract (tr)

**Amaç:**

Son yıllarda, talasemi majör olgularının evlilik ve gebelik oranları giderek artmaktadır. Bu çalışmanın amacı, talasemi hastalarının fertilite oranlarının araştırılması ve anne ve bebek sağlığı açısından gebelik sonuçlarının değerlendirilmesidir.

**Gereç ve Yöntemler:**

Bu gözlemsel çalışmada; majör hemoglobinopatili hastalar; evlenme ve çocuk sahibi olma oranları, yardımcı üreme tekniklerine gereksinimleri, demir statüleri ve gebelik komplikasyonları açısından değerlendirilmiştir.

**Bulgular:**

On yedi talasemik kadın hasta, toplam 21 sağlıklı bebek doğurmuştur. Hastaların üçte biri bebek sahibi olabilmek için yardımcı üreme tekniğine ihtiyaç duymuştur. Talasemi majör olgularının serum ferritin değerleri hamileliğin başında ortalama 1203±1206 ug/L saptanmış olup, hamileliğin sonunda 1880±1174 ug/L seviyesine yükselmiştir. Tüm bebekler halen hayatta ve sağlıklıdırlar.

**Sonuç:**

Talasemi olgularında hamilelik süreci yakın takip ve multidisipliner yaklaşım ile beraber güvenli olarak geçirilebilecektir.

## Introduction

Until the new millennium, many medical and social barriers such as limited life expectancy resulting from iron-induced cardiac disease [1,2] and significant morbidities particularly resulting from endocrine complications [1,3,4,5,6] have been main factors in the negative attitudes towards starting a family in the thalassemic population. However, therapeutic advances in the management of thalassemia have significantly improved the quality of life and life expectancy in the past two decades [7,8,9,10,11,12,13] and have consequently encouraged the thalassemic population to marry and have children. This study was conducted to assess the current tendency towards marriage among patients with thalassemia, the reproductive rate of those who wish to have children, and the course of pregnancies with respect to maternal and infant outcomes in one of the largest thalassemia centers of Turkey.

## Materials and Methods

One hundred and eighty-four patients (108 females, 76 males) with thalassemia aged above 18 years old were included in this observational study. All male and female patients who wished to have children but suffered from hypogonadotropic hypogonadism (HH) were referred to an infertility clinic. Female patients were carefully assessed for the severity of iron overload by serum ferritin (SF), cardiac T2* magnetic resonance imaging (MRI), liver R2 MRI, cardiac status by echocardiography, and the presence of endocrine disturbances. The optimization of iron burden and normalized organ functions in the pre-conception period was strongly suggested. The overall rate of fertility and the course and outcome of the pregnancies were recorded. All pregnancies were followed in close collaboration with an obstetrician. A cardiac workup was performed at 3-month intervals throughout the pregnancies.

## Results

### Fertility Rate in Female and Male Thalassemia Patients

Fifty of the 184 adult patients were married. Forty-one patients (29 females and 12 males) were married to healthy partners, and nine marriages were composed of thalassemic couples. Seventeen of the 29 female patients (59%) gave birth to 21 healthy babies (three had two pregnancies, and one had twins). Conception was spontaneous in 14 (70%) and was achieved by gonadotrophin stimulation or an assisted reproductive technique (ART) in six female patients. Overall, six of 12 male patients (50%) had seven children spontaneously while the other six, who were receiving hormone replacement therapy, did not yet have a child. Although both male and female infertility was 50%, in our cohort 33% of females but none of the males with HH could have a child.

Thalassemic couples did not wish to give birth to an affected baby. However, in a couple with beta-thalassemia intermedia (TI) and S/beta-thalassemia, spontaneous fertilization occurred. Prenatal diagnosis was performed at the 12^th^ week of gestation and genetic counseling was given to the couple, who decided to give birth to an offspring with S/beta-thalassemia.

### Disease Characteristics and the Course of the Pregnancies

The baseline characteristics of pregnant patients are reported in Table 1. The average monthly red cell concentrate (RCC) consumption showed a nonsignificant increase during pregnancy compared to the pre-pregnancy period (14.5±2.4 vs. 12.7±2.4 mL/kg/month) in patients with thalassemia major (TM). Three patients with non-transfusion-dependent thalassemia (NTDT), including TI, S/beta-thalassemia, and hemoglobin H disease, received RCC transfusions of 7.7, 7.2, and 4.2 mL/kg/month, respectively, during pregnancy to maintain the pre-transfusion hemoglobin levels of ≥8 g/dL. New red cell alloantibody formation did not occur in any patients, but cross-match compatible RCC could not be provided to the patient with TI who developed multiple alloantibodies and experienced a hemolytic transfusion reaction before pregnancy. This patient was not transfused with any incompatible RCC during pregnancy. Hemoglobin levels gradually decreased to as low as 6 g/dL and were barely maintained at around 7 g/dL by erythropoietin administration during pregnancy. Ultimately, the patient delivered a healthy full-term baby.

Iron chelation therapy was immediately ceased for all pregnant patients but deferoxamine (DFO) subcutaneous infusions were initiated after the second trimester for two subjects whose SF increased over 2229 and 7199 µg/L, and one revealed a cardiac T2* of 16 ms before pregnancy.

The TM patients had slightly increased SF from baseline (1203±1206 µg/L) until the end of pregnancy (1880±1174 µg/L). None of the patients demonstrated myocardial T2* of <20 ms in the first cardiac MRI obtained after delivery.

### Delivery and Outcomes in Newborns

All patients but one underwent a cesarean section following complication-free pregnancies. An ectopic pregnancy and a pregnancy with a fetus with trisomy 21 were terminated. Intrauterine growth retardation (IUGR) was observed in the full-term offspring of two patients with thalassemia major who maintained an average pre-transfusion hemoglobin level of 9.4 g/dL during pregnancy. Four of the 21 births (19%) were preterm (33- and 34-week singletons and 30-week twins).

Four infants were admitted to the neonatal intensive care unit due to prematurity, IUGR, or pneumothorax (Table 2). All infants were breastfed for at least 3 months.

## Discussion

Although spontaneous fertility can occur in well-transfused and well-chelated patients with thalassemia, infertility mainly due to HH still remains one of the most common morbidities and obstacles for having children [9,10,11,12,13,14,15]. In our cohort, male and female fertility rates were 50%. Gestation and delivery may result in an increased cardiac load and together with chronic hypoxia and myocardial iron deposition may aggravate cardiac dysfunction in female patients with thalassemia [10,16,17]. Severe anemia can also be a risk factor for gestational hypertension [18]. As suggested in previous studies [19,20], we assessed organ function in female patients who wished to conceive, and only those with normal cardiac function and well-controlled iron overload were encouraged to conceive. Under these conditions, cardiac health did not deteriorate in any patient during pregnancy, and all deliveries were safely performed.

Because of the potential teratogenicity of chelators, the use of chelation therapy during pregnancy has remained controversial. The current standard of practice is to cease any chelation therapy when pregnancy is established [21,22,23]. Only DFO chelation may be restarted after the first trimester when the benefits outweigh the risks of excess iron [24,25,26,27,28,29]. In our cohort, three pregnant patients received DFO after the second trimester and delivered healthy babies with no specific hearing or visual defects.

It is suggested to maintain the pre-transfusion hemoglobin at ≥10 g/dL during pregnancy in patients with thalassemia [16,30,31,32]. We have followed the current clinical practice in TM patients but have been cautious of potential risks of alloimmunization in patients with NTDT. In the latter group, the pre-transfusion hemoglobin was maintained at ≥8 g/dL.

In accordance with other reports, the majority of our patients delivered via cesarean section [33,34,35]. The prevalence of fetal and maternal complications including miscarriages, IUGR, premature labor, and even fetal death is reported to be higher in thalassemic females compared to the normal population [36,37,38]. In our cohort, premature birth was observed in 19% of the deliveries, which was considerably higher than the rate of premature spontaneous live births (6.9%) in the Turkish registry [39].

## Conclusion

Male and female thalassemic patients may conceive spontaneously, or conception may be achieved by ART. Pregnancy in patients with thalassemia can be safely managed with remarkably positive outcomes for both the mother and infant under the supervision of a multidisciplinary team.

## Figures and Tables

**Table 1:**
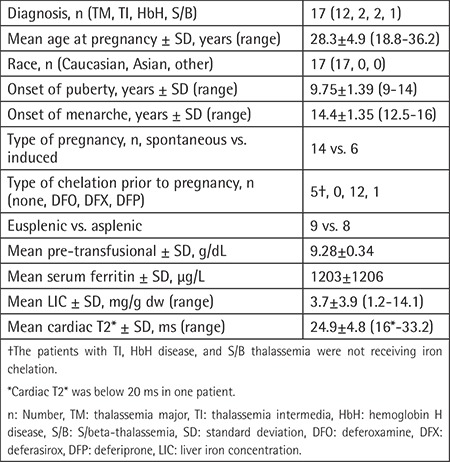
Baseline characteristics of pregnant patients with thalassemia.

**Table 2:**
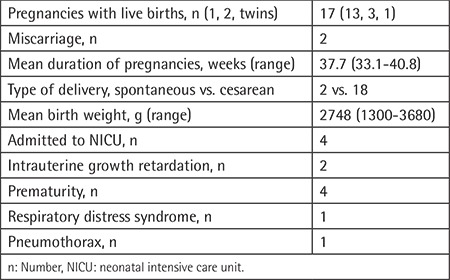
Delivery and newborn outcomes.
